# Poster 1000: Efficacy and safety of specific sublingual immunotherapy with carbamylated allergoid tablets of ragweed pollen: a dose-ranging study

**DOI:** 10.1186/1939-4551-7-S1-P1

**Published:** 2014-02-03

**Authors:** Enrico Compalati, Claudio Ortolani, Elide Pastorello, Gianna Moscato, Daniele Berra, Anna Tosi, Marina Mauro

**Affiliations:** 1 University of Genoa, IRCCS AOU San Martino-IST, Allergy and Respiratory Diseases Clinic, Genoa, Italy; 2 Allergy Unit. Istituto Sacra Famiglia. Casa di Cura Ambrosiana, Cesano Boscone, Italy; 3 Allergology and Immunology Unit. Niguarda Ca' Granda Hospital, Milan, Italy; 4 Allergy and Immunology Unit. Fondazione Salvatore Maugeri, Institute of Research and Care, Scientific Institute of Pavia, Pavia, Italy; 5 U.O. di Broncopneumologia. Azienda Ospedaliera Osp. Di Circolo di Busto Arsizio, Busto Arsizio, Italy; 6 Allergy Unit. A.O. Ospedale Civile di Legnano, Legnano, Italy; 7 P.O. Poliambulatori di via Napoleona. A.O. Ospedale Sant’Anna, Como, Italy

## Background

Carbamylated allergoids are chemically modified extracts developed to reduce the IgE binding activity and consequently improve the SLIT tolerability. This study was designed to compare the efficacy and safety of three different daily dosages of carbamylated allergoid tablets in patients allergic to ragweed pollen.

## Methods

A prospective multicenter double-blind randomized dose-ranging study (EudraCT number 2011-004522-10) was conducted following GCP rules in patients with history of ragweed-related moderate-to-severe allergic rhinoconjunctivitis for at least 2 years, with or without controlled seasonal allergic asthma, and positive response to allergen specific nasal provocation test (NPT). Before the 2013 ragweed pollen season adult patients were assigned to different daily dosages (300-1000-2000 UA) of tablets of ragweed pollen carbamylated extract given for 4 months. The primary end-point was the individual change in the response to NPT before and after the treatment (allergic symptoms and threshold provocative dose to identify improved, unchanged, worsened subjects). Secondary end-points included the analysis of this improvement considering the severity level (depending on symptoms score and provocative dose), the change of nasal peak inspiratory flow (NPIF) in response to NPT, and the incidence of adverse events.

## Results

Seventy-three subjects were enrolled and 52 were randomized (1:1:1) and treated. The proportion of improved patients was 77%, 88% and 81% in the 300 UA, 1000UA and 2000 UA groups respectively. A progressive trend to increased efficacy with flattening of the slope (mean severity level improvement: +1.38, +1.71 and +1.90 respectively) and a statistically significant difference between 300 UA and 2000 UA (p = 0.0187) were found. NPIF improved in all groups at the end of the study without significant differences among treatments (p = 0.41). One probable treatment-related AE occurred in group 1, 3 probable and 1 certain (mild) in group 2, 3 possible and 1 certain (moderate) in group 3. No serious treatment-related AEs occurred.

## Conclusions

The three daily dosages provided improvement in the response to the allergen specific NPT in a large proportion of patients. The group receiving 1000 UA showed the highest percentage of improved patients. The mean improvement and the incidence of treatment-related AEs suggest that this dosage appears to be the ideal dose of carbamylated allergoid for treating ragweed pollen allergic patients.

